# QuickStats

**Published:** 2013-04-19

**Authors:** Gulnur Freeman, Patricia F. Adams

**Figure:**
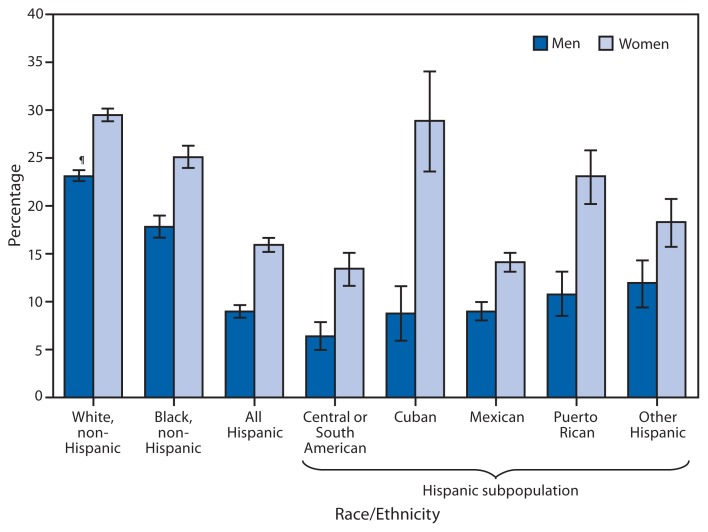
Percentage of Adults Ever Told They Have Some Form of Arthritis or a Related Condition,^*^ by Sex, Race/Ethnicity, and Hispanic^†^ Subpopulation — National Health Interview Survey, United States, 2011^§^ ^*^ Based on a survey question that asked respondents, “Have you ever been told by a doctor or other health professional that you have some form of arthritis, rheumatoid arthritis, gout, lupus, or fibromyalgia?” Those that answered yes were classified as having an arthritis diagnosis. Unknowns were not included in the denominators when calculating percentages. ^†^ Persons of Hispanic ethnicity might be of any race or combination of races. Non-Hispanic persons are those who are not of Hispanic ethnicity, regardless of race. ^§^ Estimates are based on household interviews of a sample of the noninstitutionalized U.S. civilian population. ^¶^ 95% confidence interval.

During 2011, in each racial/ethnic group considered, women were more likely than men to have been told by a doctor or other health professional that they have arthritis or a related condition. Among men and women, Hispanic adults were less likely than non-Hispanic white and non-Hispanic black adults to have been told that they have arthritis. Among Hispanic subpopulations, considerable variation occurred, with notably higher rates for Cuban and Puerto Rican women.

**Source:** National Health Interview Survey, 2011 sample adult core component. Available at http://www.cdc.gov/nchs/nhis.htm.

